# Associations between perceived care quality, self-care behaviors, and glycemic control in Chinese adults with type 2 diabetes under the national essential public health services program

**DOI:** 10.1186/s12889-024-19538-y

**Published:** 2024-07-23

**Authors:** Dianjiang Li, Enchun Pan, Zhongming Sun, Jinbo Wen, Ming Su, Ming Wu, Jian Su, Jinyi Zhou, Hong Fan, Chong Shen

**Affiliations:** 1 Department of Social Medicine and Health Education, School of Public Health, Nanjing Medical University, Nanjing, China; 2 Department of Chronic Disease Prevention and Control, Huai’an City Center for Disease Control and Prevention, Huai’an, China; 3 Huai’an District Center for Disease Control and Prevention, Huai’an, China; 4 Department of Chronic Disease Prevention and Control, Jiangsu Provincial Center for Disease Control and Prevention, Nanjing, China

**Keywords:** Type 2 diabetes, Perceived care quality, Self-care behaviors, Glycemic control, China

## Abstract

**Background:**

The rising prevalence of Type 2 diabetes (T2D) in China poses a critical health challenge, necessitating effective management strategies. The National Essential Public Health Services Program (NEPHSP), initiated in 2009, focuses on equitable access to health services, including T2D management. This study investigates the associations between perceived care quality, self-care behaviors, and glycemic control in Chinese adults with T2D under NEPHSP, particularly examining the mediating role of self-care behaviors.

**Methods:**

Conducted from April to November 2020 in Huai’an City, Jiangsu Province, this study involved 1,577 T2D patients enrolled in NEPHSP. We assessed perceived care quality using the Patient Assessment of Chronic Illness Care (PACIC) scale and developed a comprehensive self-care behavior score, covering nine essential health practices. Glycemic control was evaluated using HbA1c levels. Linear regression models were used to explore these associations, adjusting for demographic and clinical factors, while causal mediation analyses examined the role of intermediate variables.

**Results:**

Higher PACIC scores significantly correlated with improved self-care behaviors (β = 0.294, 95% CI: 0.233 to 0.354) and were negatively associated with HbA1c levels (β=-0.109, 95% CI: -0.192 to -0.026). The self-care behavior score inversely related to HbA1c levels (β=-0.197, 95% CI: -0.263 to -0.132). Notably, self-care behaviors mediated 50.41% (*P* < 0.05) of the effect of perceived care quality on HbA1c levels.

**Conclusions:**

This study demonstrates a substantial association between perceived care quality and better glycemic control in Chinese adults with T2D under NEPHSP, with self-care behaviors playing a crucial mediating role. These findings suggest that patient-centered care and comprehensive self-care practices are essential for effective T2D management within NEPHSP.

**Supplementary Information:**

The online version contains supplementary material available at 10.1186/s12889-024-19538-y.

## Background

Type 2 diabetes (T2D) poses a significant global health challenge [1], with its incidence notably rising in China [2]. Currently, diabetes prevalence is expected to surpass 11.2%, affecting more than 129 million Chinese adults [3]. This increase heightens the risk of cardiovascular mortality, emphasizing the urgency of improved management of T2D [4]. Effective management depends crucially on continuous monitoring of glycemic control, primarily assessed through glycated hemoglobin (HbA1c) levels [4]. Monitoring HbA1c is vital as it serves as a reliable indicator of long-term glycemic control and aids in predicting the risk of diabetic complications [4].

To tackle this challenge and enhance public health outcomes, the Chinese government initiated the National Essential Public Health Services Program (NEPHSP) in 2009 [4–6]. The NEPHSP, with its focus on primary healthcare, aims to provide equitable access to essential health services nationwide [7]. It offers a broad spectrum of services, including health assessments and tailored interventions, with a special emphasis on T2D management [6, 7]. Crucially, this approach fosters a collaborative effort between healthcare professionals and patients, which is vital for the effective management of T2D [4, 6].

Within the framework of the NEPHSP, ensuring high-quality, patient-centered care for T2D is essential [4, 7]. The Patient Assessment of Chronic Illness Care (PACIC) is globally recognized for evaluating the quality of care from patients’ perspectives [8–10]. Extensively validated for a range of chronic conditions, including diabetes [11], studies consistently link PACIC scores to enhanced patient satisfaction and improved health outcomes [9, 11]. However, the specific relationship between PACIC scores and glycemic control in diabetes has not been thoroughly investigated. While several studies have attempted to explore this association, their findings are mixed; some report a positive impact of high PACIC scores on glycemic control, while others find no significant effect [12–14]. This inconsistency highlights a significant gap in the research, necessitating further exploration. Given the critical role of glycemic control in preventing severe diabetes-related complications, understanding how PACIC scores influence glycemic control is essential for assessing the effectiveness of patient-centered care strategies in the management of T2D.

Self-care is a critical element in managing T2D within the NEPHSP, underscoring the importance of patient participation in their health management [15]. Key self-care practices, including regular glucose monitoring, medication adherence, lifestyle modifications, and health check-ups, are essential components of effective diabetes management [15, 16]. These practices are vital for improving health outcomes, with substantial evidence supporting their positive impact on glycemic control [16, 17]. Research indicates that these self-care behaviors often correlate with each other and may exhibit interactive effects [18], suggesting a more complex dynamic than previously understood. The cumulative impact of these multiple behaviors on glycemic control, especially considering the intricate nature of glucose metabolism in T2D, has not been thoroughly investigated. This gap presents a significant opportunity for public health research, particularly in understanding how these combined behaviors might translate into improved health outcomes [18]. Therefore, it is imperative to develop a self-care behavior score to evaluate the impact of combined self-care behaviors on glycemic control. While some studies have explored the relationship between perceived care quality and self-care behaviors [9, 11, 19], the mediating role of self-care in linking perceived care quality and glycemic control is not clearly established.

In light of this context, our study endeavors to examine the relationship between perceived diabetes care quality, comprehensive self-care behaviors, and glycemic control in Chinese adults with T2D under NEPHSP. We also seek to investigate the self-care behavior score’s mediating role in the relationship between perceived care quality and glycemic control.

## Methods

### Study design and participants

This cross-sectional analysis is part of a community-based study conducted in Huai’an City, Jiangsu Province, China, under the NEPHSP focusing on the management of T2D. Recruitment for the Comprehensive Research on the Prevention and Control of Diabetes (CRPCD) project commenced in 2013. Using stratified cluster sampling, as previously detailed [20], we selected the Qinghe and Huai’an districts from the city’s eight districts. Within these, 26 townships were randomly chosen, and one community health service center per township was selected. All registered patients at these centers undergoing T2D management were included in the 2013 baseline survey. Data collection occurred from April to November 2020, during which 9,807 participants, initially recruited in 2013, completed the follow-up survey and provided blood samples. Of these, 2,186 were randomly chosen for evaluating the quality of diabetes care using the PACIC. Trained professionals conducted face-to-face interviews to collect the data.

This analysis was limited to participants who were taking glucose-lowering medication (*n* = 1,731). Participants without complete self-care behavior data (*n* = 36) or those diagnosed with conditions potentially influencing self-care practices, such as obstructive sleep apnea hypopnea syndrome (*n* = 23), diabetic lower limb ulcers or gangrene (*n* = 30), diabetic foot (*n* = 15), or cancer (*n* = 50), were excluded. As a result, 1,577 participants remained for the current analysis. Sample size determination, using G*power software version 3.1.9.7 [21], assumed 12 predictors in a regression model, a medium effect size of 0.15, a power of 0.80, and a significance level of 0.05. This calculation indicated a minimum required sample size of 127, a threshold comfortably exceeded by the study, thus ensuring robust statistical power.

### T2D and glycemic control

T2D is defined by fasting plasma glucose levels ≥ 7.0 mmol/L, an HbA1c level ≥ 6.5%, or a self-reported history of T2D, excluding type 1 diabetes cases [15]. HbA1c is a crucial diagnostic tool, reflecting average blood glucose levels over the previous two to three months without being affected by daily dietary intake, thus offering advantages over oral glucose tolerance tests and glucometer readings [22]. In this study, HbA1c values were used as the measure of glycemic control.

Blood samples were procured either 8 h post the last meal or following an overnight fast. HbA1c measurements were obtained using high-performance liquid chromatography, with all analyses conducted by King Med Diagnostics (Jiangsu Cultural Industrial Park, Nanjing, China).

### Perceived quality of care

The PACIC scale, comprising 20 items, was employed to evaluate perceived care quality in T2D patients [8, 9]. This scale, previously validated for assessing health management in this patient group [13, 23], involves participants rating the frequency of encountering specific scenarios over the past six months on a 5-point scale (1 indicating “almost never” and 5 “almost always”). The PACIC includes five subscales: patient activation, delivery system/practice design, goal setting/tailoring, problem-solving/contextual, and follow-up/coordination. Scores for both the overall scale and individual subscales range up to 5, with higher scores indicating better perceived quality of care. In this study, the Cronbach’s alpha for the subscales ranged from 0.80 to 0.91, with an overall scale reliability of 0.97.

### Self-care behaviors

Informed by prior research and Chinese diabetes prevention and management guidelines [15], we constructed a self-care behavior score, encompassing no current smoking, no alcohol consumption, healthy diet, regular physical activity, less sedentary behavior, adequate sleep duration, regular self-monitoring of blood glucose, adequate medication adherence, and regular health check-ups (Supplementary Table S1).

“No current smoking” was referred to individuals either having smoked fewer than 100 cigarettes throughout their life or having ceased their smoking habits by the time of assessment. Similarly, “no alcohol consumption” was referred to lifelong abstainers or those who had stopped alcohol intake by the assessment time. A “healthy diet” was qualified by the daily consumption of both fruits and vegetables [24].

Leisure-time physical activity (LTPA) was assessed using the Chinese version of the Global Physical Activity Questionnaire (GPAQ-C), which has demonstrated good validity and reliability [25]. Participants detailed their typical activities, classifying them into vigorous (e.g., swimming, running, aerobic exercise) or moderate (e.g., ball games, walking, gymnastics, folk dancing, Tai-Chi, qigong) intensity categories, and provided information regarding the frequency and weekly duration of these activities. Subsequently, the total moderate-to-vigorous LTPA was quantified in metabolic equivalent of task (MET) minutes per week, employing a calculation that multiplied the duration of activities by the respective MET scores—4.0 MET for moderate and 8.0 MET for vigorous intensity activity, in alignment with GPAQ guidelines. “Regular physical activity” was defined as engagement in at least 75 min of vigorous or 150 min of moderate LTPA per week (or an equivalent combination), equivalent to ≥ 600 MET-minutes/week [26].

Participants also detailed their sedentary behaviors, counting time spent commuting, working, watching TV, using electronic devices, and engaging in passive activities like reading or playing games. “Less sedentary behavior” was attributed to those reporting under 4 h daily. “Adequate sleep duration” was determined by nightly rest periods of 6–8 h [27], and “Regular self-monitoring of blood glucose” applied to those conducting at least weekly blood glucose self-checks.

Medication adherence was assessed through four questions: “Do you ever forget to take your glucose-lowering medications?“, “Have you been persuaded by non-medical personnel to alter your glucose-lowering regimen?“, “Do you ever skip your glucose-lowering medications when you feel well?“, and “Have you adjusted your glucose-lowering medication in dosage or type due to illness without consulting a doctor?“. Participants rated each question on a scale of 0 to 2 points indicating ‘always’, ‘sometimes’, or ‘never’. A cumulative score of 8 denoted “adequate medication adherence”. Adherence to health check-ups was similarly assessed with questions such as: “Do you ever miss scheduled health check-ups?“, “Do you ever seek a check-up only when feeling unwell?“, “Do you ever skip health check-ups when feeling well?“, and “Have you missed check-ups for over three months?“. A score of 8 denoted “regular health check-ups”.

The scoring system was designed to reflect the number of self-care activities a participant engaged in, with each participant receiving a point for each criterion met. This resulted in total scores ranging from 0 to 9, with higher scores indicating more optimal self-care. This method aligns with common practices in health behavior research [28–30], where equal weight is typically given to various lifestyle behaviors.

### Other covariates

Data on sociodemographic and clinical characteristics were obtained through a standardized questionnaire (Supplementary Table S1). Marital status was classified as either married or non-married (including single, separated, divorced, and widowed). Educational attainment was classified into three tiers: primary school or lower, junior school, and senior school or higher. Occupational status was categorized as either employed, unemployed, or retired. Annual household income was divided into three categories: <10, 10–100, or ≥ 100 *thousand Yuan*. Types of glucose-lowering medications comprised oral antihyperglycemic agents, insulin, or both.

We identified individuals with documented histories of hypertension or dyslipidemia based on professional medical records. Participants also reported specific microvascular complications, including nephropathy, retinopathy, and neuropathy, and macrovascular complications such as coronary artery disease, stroke, and peripheral arterial disease, all corroborated by official medical documentation.

### Statistical methods

Continuous variables with skewed distributions were presented as medians (interquartile ranges, IQRs), whereas categorical variables were described using frequencies (percentages). We explored the associations between the overall PACIC score, its subscales, and either the self-care behavior score or HbA1c levels using linear regression. This method also elucidated the relationships between the self-care behavior score, individual self-care behaviors, and HbA1c levels. Logistic regression was utilized to investigate the associations between the overall PACIC score and each specific self-care behavior. Both linear and logistic regressions employed three models: Model 1, Unadjusted; Model 2, Adjusted for age (continuous), gender, marital status, educational attainment, occupational status, annual household income, and glucose-lowering medication types; and Model 3, Further adjusted for history of hypertension, history of dyslipidemia, and the number of micro- and macrovascular complications, accounting for potential changes in self-care post-diagnosis and the influence of extensive medical services from general practitioners on perceived care quality.

Utilizing a counterfactual framework [31], causal mediation analyses were conducted to investigate the potential mediation of the self-care behavior score in the association of overall PACIC score, and its subscales, with HbA1c. The analyses encompassed estimations for the natural direct effect (NDE: the influence of the PACIC score/subscales on HbA1c without the self-care behavior score), natural indirect effect (NIE: influence of the PACIC score/subscales on HbA1c through the self-care behavior score), total effect (TE: summation of NDE and NIE), and percentage mediated (PM: the portion of TE mediated through the self-care behavior score). Utilizing the PROC CAUSALMED procedure in SAS [32], these estimations were facilitated, offering causal mediation effects and confidence intervals (*CI*s) derived from maximum likelihood estimates, while 1000 bootstrap resamples enhanced the computation of *CI*s for causal mediation effects [33]. The models were adjusted for potential confounders, including age (continuous), gender, marital status, educational attainment, occupational status, annual household income, glucose-lowering medication types, history of hypertension, history of dyslipidemia, and the number of micro- and macrovascular complications.

To test the robustness of our mediation findings, sensitivity analyses were implemented. Initially, participants who ceased smoking or drinking due to familial persuasion or financial constraints were excluded. Subsequently, individuals with micro- and macrovascular complications were excluded to mitigate potential reverse causation.

All statistical evaluations were conducted using SAS version 9.4 (SAS Institute, Cary, NC). A two-tailed *P*-value < 0.05 denoted statistical significance.

## Results

### Characteristics of participants

Table 1 presents the baseline characteristics of 1,577 participants. The participants’ median age was 68 years (IQR: 62–73 years), with those aged ≥ 65 years representing 65.50%. Most had an education level of primary school or lower (74.83%), were female (63.73%), married (84.27%), prescribed oral antihyperglycemic agents (74.89%), and had a history of hypertension (53.52%). Additionally, 26.25% indicated an annual household income of less than 10 *thousand Yuan*, 32.78% had a history of dyslipidemia, and 19.47% presented with micro- or macrovascular complications. The median HbA1c was 7.9 (IQR: 6.8–9.5), with 27.58% achieving HbA1c values of ≤ 7.0%. The median self-care behavior score was 4 (IQR: 3–5). Among participants, 80.34% had no current smoking, 83.45% reported no alcohol consumption, 16.04% maintained a healthy diet, 5.52% engaged in regular physical activity, 70.77% showed less sedentary behavior, 58.91% achieved adequate sleep duration, 17.95% regularly monitored their blood glucose, 52.50% adhered to their medication, and 50.10% had regular health check-ups. The overall median PACIC score was 3.15 (IQR: 2.35–4.10). PACIC subscale medians were as follows: 3.33 (IQR: 2.33–4.33) for patient activation, 3.67 (IQR: 3.00-4.67) for delivery system/practice design, 3.20 (IQR: 2.20–4.20) for goal setting/tailoring, 3.00 (IQR: 2.00-4.25) for problem-solving/contextual, and 3.00 (IQR: 2.00–4.00) for follow-up/coordination.

**Table 1:** Baseline characteristics of the study participants(*n* = 1,577)

Variables	*n*(%) or median (IQR)	
**Age (years)**	68(62,73)	
< 65	544(34.50)	
≥ 65	1033(65.50)	
**Gender**		
Male	572(36.27)	
Female	1005(63.73)	
**Education attainment**		
Primary school and below	1180(74.83)	
Junior school	272(17.25)	
Senior school and above	125(7.93)	
**Marital status**		
Married	1329(84.27)	
Non-married	248(15.73)	
**Annual household income (** ***thousand Yuan*** **)**		
< 10	414(26.25)	
10–100	624(39.57)	
≥ 100	539(34.18)	
**Occupational status**		
Employed	755(47.88)	
Unemployed	660(41.85)	
Retired	162(10.27)	
**Glucose-lowering medication types**		
Oral antihyperglycemic agents	1181(74.89)	
Insulin	204(12.94)	
Both	192(12.18)	
**History of hypertension**		
Yes	844(53.52)	
No	733(46.48)	
**History of dyslipidemia**		
Yes	517(32.78)	
No	1060(67.22)	
**No. of micro- and macrovascular complications**		
0	1270(80.53)	
1	92(5.83)	
2	185(11.73)	
3	18(1.14)	
4	9(0.57)	
5	2(0.13)	
6	1(0.06)	
**Self-care behavior score**	4(3,5)	
No current smoking	1267(80.34)	
No alcohol consumption	1316(83.45)	
Healthy diet	253(16.04)	
Regular physical activity	87(5.52)	
Less sedentary behavior	1116(70.77)	
Adequate sleep duration	929(58.91)	
Regular self-monitoring of blood glucose	283(17.95)	
Adequate medication adherence	828(52.50)	
Regular health check-ups	790(50.10)	
**PACIC**		
Patient activation	3.33(2.33,4.33)	
Delivery system/practice design	3.67(3.00,4.67)	
Goal setting/tailoring	3.20(2.20,4.20)	
Problem-solving/contextual	3.00(2.00,4.25)	
Follow-up/coordination	3.00(2.00,4.00)	
Overall PACIC score	3.15(2.35,4.10)	
**HbA1c (%)**	7.9(6.8–9.5)	
< 7	435(27.58)	
7–8	361(22.89)	
≥ 8	781(49.52)	

*IQR* interquartile range, *PACIC* Patient Assessment of Chronic Illness Care

### Association between PACIC and the self-care behavior score

After controlling for age, gender, marital status, educational attainment, occupational status, annual household income, glucose-lowering medication types, history of hypertension, history of dyslipidemia, and the number of micro- and macrovascular complications, several significant associations were observed in multivariate linear regression models. Specifically, the Overall PACIC score (β = 0.294, 95% CI: 0.233 to 0.354), patient activation (β = 0.232, 95% CI: 0.180 to 0.284), delivery system/practice design (β = 0.235, 95% CI: 0.169 to 0.301), goal setting/tailoring (β = 0.252, 95% CI: 0.197 to 0.307), problem-solving/contextual (β = 0.244, 95% CI: 0.190 to 0.298), and follow-up/coordination (β = 0.256, 95% CI: 0.201 to 0.311) demonstrated significant associations with the self-care behavior score (Table 2). Additionally, the overall PACIC score was significantly associated with regular blood glucose monitoring (OR = 1.149, 95% CI: 1.018 to 1.297), adequate medication adherence (OR = 1.415, 95% CI: 1.290 to 1.553), and regular health check-ups (OR = 1.775, 95% CI: 1.609 to 1.958) in multivariable logistic regression models (Supplementary Table S2).

**Table 2:** Association of the overall PACIC score and its subscales with the self-care behavior score by using linear regression models(*n* = 1,577)

PACIC	Model 1			Model 2			Model 3	
	β (95% CI)	*P* value		β (95% CI)	*P* value		β (95% CI)	*P* value
Patient activation	0.237(0.184,0.289)	< 0.001		0.232(0.180,0.284)	< 0.001		0.232(0.180,0.284)	< 0.001
Delivery system/practice design	0.248(0.182,0.314)	< 0.001		0.233(0.167,0.299)	< 0.001		0.235(0.169,0.301)	< 0.001
Goal setting/tailoring	0.250(0.195,0.305)	< 0.001		0.252(0.197,0.307)	< 0.001		0.252(0.197,0.307)	< 0.001
Problem-solving/contextual	0.250(0.196,0.304)	< 0.001		0.246(0.192,0.299)	< 0.001		0.244(0.190,0.298)	< 0.001
Follow-up/coordination	0.258(0.203,0.313)	< 0.001		0.259(0.204,0.313)	< 0.001		0.256(0.201,0.311)	< 0.001
Overall PACIC score	0.297(0.237,0.358)	< 0.001		0.294(0.234,0.354)	< 0.001		0.294(0.233,0.354)	< 0.001

Model 1: unadjusted

Model 2: adjusted for age (continuous), gender, marital status, educational attainment, occupational status, annual household income, and glucose-lowering medication types

Model 3: further adjusted for history of hypertension, history of dyslipidemia, and the number of micro- and macrovascular complications

*PACIC* Patient Assessment of Chronic Illness Care, *CI* confidence interval

### Association between PACIC and HbA1c

In multivariable linear regression models, after adjusting for age, gender, marital status, educational attainment, occupational status, annual household income, glucose-lowering medication types, history of hypertension, history of dyslipidemia, and the number of micro- and macrovascular complications, the Overall PACIC score (β=-0.109, 95% CI: -0.192 to -0.026), patient activation (β=-0.101, 95% CI: -0.172 to -0.031), problem-solving/contextual (β=-0.081, 95% CI: -0.154 to -0.007), and follow-up/coordination (β=-0.124, 95% CI: -0.199 to -0.049) were significantly negatively associated with HbA1c (Table 3). However, no significant associations were observed between delivery system/practice design (β=-0.063, 95% CI: -0.152 to 0.026) or goal setting/tailoring (β=-0.073, 95% CI: -0.149 to 0.002) with HbA1c (Table 3).

**Table 3:** Association of the overall PACIC score and its subscales with HbA1c by using linear regression models(*n* = 1,577)

PACIC	Model 1			Model 2			Model 3	
	β (95% CI)	*P* value		β (95% CI)	*P* value		β (95% CI)	*P* value
Patient activation	-0.086(-0.156,-0.015)	0.018		-0.100(-0.171,-0.030)	0.005		-0.101(-0.172,-0.031)	0.005
Delivery system/practice design	-0.043(-0.132,0.046)	0.342		-0.062(-0.150,0.027)	0.171		-0.063(-0.152,0.026)	0.167
Goal setting/tailoring	-0.058(-0.133,0.017)	0.132		-0.072(-0.147,0.003)	0.062		-0.073(-0.149,0.002)	0.058
Problem-solving/contextual	-0.068(-0.141,0.005)	0.069		-0.079(-0.152,-0.006)	0.033		-0.081(-0.154,-0.007)	0.032
Follow-up/coordination	-0.114(-0.189,-0.039)	0.003		-0.122(-0.197,-0.048)	0.001		-0.124(-0.199,-0.049)	0.001
Overall PACIC score	-0.092(-0.175,-0.010)	0.029		-0.107(-0.190,-0.025)	0.011		-0.109(-0.192,-0.026)	0.010

Model 1: unadjusted

Model 2: adjusted for age (continuous), gender, marital status, educational attainment, occupational status, annual household income, and glucose-lowering medication types

Model 3: further adjusted for history of hypertension, history of dyslipidemia, and the number of micro- and macrovascular complications

*PACIC* Patient Assessment of Chronic Illness Care, *CI* confidence interval

### Association between the self-care behavior score and HbA1c

In multivariable linear regression models, when controlling for age, gender, marital status, educational attainment, occupational status, annual household income, glucose-lowering medication types, history of hypertension, history of dyslipidemia, and the number of micro- and macrovascular complications, significant associations with HbA1c were observed for the self-care behavior score (β=-0.197, 95% CI: -0.263 to -0.132), no current smoking (β=-0.590, 95% CI: -0.827 to -0.353), adequate sleep duration (β=-0.247, 95% CI: -0.437 to -0.057), regular self-monitoring of blood glucose (β=-0.369, 95% CI: -0.620 to -0.118), and regular health check-ups (β=-0.405, 95% CI: -0.591 to -0.219) (Table 4).

**Table 4:** Association of the self-care behavior score and individual self-care behaviors with HbA1c by using linear regression models(*n* = 1,577)

Self-care behaviors	Model 1			Model 2			Model 3	
	β (95% CI)	*P* value		β (95% CI)	*P* value		β (95% CI)	*P* value
No current smoking	-0.531(-0.765,-0.297)	< 0.001		-0.587(-0.824,-0.351)	< 0.001		-0.590(-0.827,-0.353)	< 0.001
No alcohol consumption	0.072(-0.179,0.324)	0.573		-0.008(-0.285,0.269)	0.956		0.001(-0.277,0.279)	0.994
Healthy diet	-0.248(-0.503,0.006)	0.056		-0.213(-0.468,0.042)	0.102		-0.221(-0.477,0.034)	0.090
Regular physical activity	0.039(-0.371,0.449)	0.852		0.094(-0.318,0.505)	0.655		0.079(-0.333,0.492)	0.706
Less sedentary behavior	-0.166(-0.372,0.039)	0.113		-0.172(-0.379,0.035)	0.103		-0.177(-0.384,0.031)	0.095
Adequate sleep duration	-0.229(-0.419,-0.039)	0.018		-0.247(-0.437,-0.057)	0.011		-0.247(-0.437,-0.057)	0.011
Regular self-monitoring of blood glucose	-0.238(-0.481,0.006)	0.056		-0.358(-0.608,-0.108)	0.005		-0.369(-0.620,-0.118)	0.004
Adequate medication adherence	-0.124(-0.312,0.063)	0.194		-0.123(-0.310,0.064)	0.197		-0.127(-0.314,0.060)	0.183
Regular health check-ups	-0.419(-0.605,-0.232)	< 0.001		-0.411(-0.596,-0.225)	< 0.001		-0.405(-0.591,-0.219)	< 0.001
Self-care behavior score	-0.180(-0.245,-0.115)	< 0.001		-0.195(-0.260,-0.130)	< 0.001		-0.197(-0.263,-0.132)	< 0.001

Model 1: unadjusted

Model 2: adjusted for age (continuous), gender, marital status, educational attainment, occupational status, annual household income, and glucose-lowering medication types

Model 3: further adjusted for history of hypertension, history of dyslipidemia, and the number of micro- and macrovascular complications

*CI* confidence interval

### Mediation effect of the self-care behavior score

In the causal mediation analysis, adjustments were made for age, gender, marital status, educational attainment, occupational status, annual household income, glucose-lowering medication types, history of hypertension, history of dyslipidemia, and the number of micro- and macrovascular complications. Table 5 illustrates that the self-care behavior score significantly mediated the relationships between the overall PACIC score, its subscales (patient activation, problem-solving/contextual, follow-up/coordination), and HbA1c levels. The proportions of these mediation effects relative to the TE were 50.41% (*P* < 0.05), 42.57% (*P <* 0.05), 57.74% (*P <* 0.05), and 37.48% (*P <* 0.01), respectively.

**Table 5:** Mediation analyses for the association of the overall PACIC score and its subscales with HbA1c through the self-care behavior score(*n* = 1,577)

PACIC	TE, β (95% CI)	NDE, β (95% CI)	NIE, β (95% CI)	PM, % (95% CI)
Patient activation	-0.101(-0.172,-0.031)**	-0.058(-0.130,0.013)	-0.043(-0.061,-0.025)**	42.57(9.09,76.04)*
Delivery system/practice design	-0.063(-0.151,0.026)	-0.017(-0.106,0.072)	-0.046(-0.066,-0.026)**	73.15(-32.12,178.42)
Goal setting/tailoring	-0.073(-0.148,0.002)	-0.025(-0.101,0.052)	-0.049(-0.068,-0.029)**	66.46(-5.13,138.05)
Problem-solving/contextual	-0.081(-0.154,-0.008)*	-0.034(-0.108,0.040)	-0.047(-0.066,-0.027)**	57.74(1.85,113.62)*
Follow-up/coordination	-0.124(-0.199,-0.050)**	-0.078(-0.154,-0.002)**	-0.047(-0.066,-0.027)**	37.48(10.84,64.11)**
Overall PACIC score	-0.109(-0.191,-0.027)**	-0.054(-0.138,0.030)	-0.055(-0.078,-0.032)**	50.41(8.36,92.45)*

Models were adjusted for age (continuous), gender, marital status, educational attainment, occupational status, annual household income, glucose-lowering medication types, history of hypertension, history of dyslipidemia, and the number of micro- and macrovascular complications

*PACIC* Patient Assessment of Chronic Illness Care, *TE* total effect, *NDE* natural direct effect, *NIE* natural indirect effect, *PM* percentage mediated, *CI* confidence interval

* *P* < 0.05; ** *P* < 0.01

In the sensitivity analyses, after excluding those who ceased smoking or drinking due to familial persuasion or financial constraints or those with micro- and macrovascular complications, the mediating effects of the self-care behavior score remained largely consistent with the primary analysis (Supplementary Table S3).

## Discussion

To our knowledge, our research is the first to uncover a complex interplay between patients’ perceptions of diabetes care quality, comprehensive self-care behaviors, and glycemic control within the context of the NEPHSP in China. Our results reveal a positive association between higher perceived care quality and improved glycemic control. Notably, combined self-care behaviors were found to mediate more than half of this relationship, independent of demographic and clinical factors.

In assessing care quality, the PACIC has demonstrated valid and reliable, reflecting the care received by Chinese adults with T2D under the NEPHSP [13]. Contrary to findings from a study in the United States, which did not find a significant link between PACIC and HbA1c levels [12], our research demonstrates a clear association. This discrepancy may stem from variations in the instruments used; the U.S. study employed a modified PACIC, which lacked validation for the original structure and showed no significant correlations with HbA1c [12]. In contrast, our study utilized the standard PACIC, consistent with other studies from Shanghai, China [13] and Queensland, Australia [14]. Notably, our analysis highlights that specific PACIC subscales - patient activation, problem-solving/contextual, and follow-up/coordination - are independently linked to improved glycemic control. This insight provides healthcare providers with actionable intelligence for interventions focusing on these care aspects to significantly improve patient outcomes.

We also discovered a significant association between a comprehensive self-care score and glycemic regulation, in line with research from the United States [34, 35] and Pakistan [17], which typically adopted narrower definitions of self-care. Particularly, behaviors like smoking cessation, adequate sleep duration, regular self-monitoring of blood glucose, and consistent health check-ups were linked to better glycemic control, echoing previous findings [17, 18, 36–38]. However, our findings diverge from earlier studies [18, 39–41] in that behaviors like alcohol abstinence, maintaining a healthy diet, regular physical activity, minimizing sedentary time, and medication adherence did not show a significant link to improved glycemic levels. This discrepancy may stem from variations in defining these behaviors or their prevalence across different populations [42]. A holistic approach to self-care, considering the complexity of glucose metabolism, may be more beneficial for individuals with T2D than focusing on isolated behaviors [18].

Further, our research highlights the impact of patients’ perceptions of care quality on enhancing self-care behaviors. We found a positive association between higher PACIC scores and improved self-care, aligning with prior studies [9, 19, 43] that linked PACIC scores with increased self-care activities, albeit using narrower definitions of self-care. Notably, our study identified a positive association between PACIC scores and all individual self-care behaviors, with the notable exception of physical activity. This exception could be attributed to the age profile of our participants, primarily aged over 65, who might face mobility challenges impacting their ability to adhere to recommended levels of physical activity.

A novel aspect of our study is the identification of self-care behaviors as mediators in the relationship between perceived quality of diabetes care and glycemic control. In contrast to a cross-sectional study in Oman, which did not observe this mediation effect possibly due to a smaller sample size of only 273 T2D patients [43], our research indicates that a composite of self-care behaviors explains significant portions of the variance in this relationship. Specifically, these behaviors account for 50.41%, 42.57%, 57.74%, and 37.48% of the variance for the overall PACIC score and its subscales (patient activation, problem-solving/contextual, and follow-up/coordination) in relation to HbA1c levels. This insight is crucial for refining intervention strategies to more effectively target these behaviors. Consequently, current healthcare interventions should be redesigned to enhance key aspects of patient-centered care such as patient activation, problem-solving skills in a diabetes context, and follow-up and coordination. By focusing on these specific areas, we could achieve more substantial improvements in glycemic control by promoting effective self-care practices among patients.

Given the NEPHSP’s emphasis on equitable access to healthcare and the importance of continuous monitoring and tailored interventions, our findings underscore the need for policy adjustments to further enhance patient-centered care. The NEPHSP’s community-based T2D management initiatives in China provide an essential platform for emphasizing self-management support and education [4, 6, 15], crucial for improving outcomes such as HbA1c levels. Health systems should integrate these insights into their patient education programs and provider training modules, particularly focusing on interventions that boost patient activation, facilitate contextual problem-solving, and improve follow-up and coordination. These strategies not only align with public health goals to reduce diabetes-related complications and enhance overall patient well-being but also are vital for managing China’s large diabetes population [2]. By establishing clear objectives and support mechanisms, healthcare administrators and policymakers can significantly improve the quality of patient-centered care, thereby helping to control the growth of comorbidities and reduce the burden on tertiary healthcare systems.

This study marks a significant advancement in developing a comprehensive self-care behavior score to examine the complex interactions among self-care behaviors, perceived diabetes care quality, and glycemic control. We formulated a score encompassing nine essential health practices, each assessed according to criteria consistent with Chinese diabetes prevention and management guidelines [15]. This alignment was crucial to ensure the score’s relevance and applicability to our specific study population in China. Contrastingly, the widely recognized Summary of Diabetes Self-Care Activities (SDSCA) by Toobert et al. [44]. comprises 13 items spanning six dimensions: diet, exercise, blood glucose testing, foot care, medication taking, and smoking. However, these dimensions do not completely conform to the recommendations of the Chinese guidelines, prompting us to devise a score better suited to our research context. Our tailored score explores the mediating effects on the relationship between perceived diabetes care quality and glycemic control, maintaining its effectiveness even when excluding patients affected by external factors or health complications.

The study faces several limitations. First, its geographical scope, limited to Huai’an City, Jiangsu Province, China, may restrict the generalizability of the findings. Second, despite employing rigorous methods, the potential for residual and unmeasured confounding variables exists. diabetes self-management education is a potential confounder for self-management behavior. Notably, data on diabetes duration was not collected due to the long-term management of the disease (over 7 years) by participants, potentially affecting their ability to accurately recall their condition’s duration. Additionally, diabetes self-management education was not considered, yet it is a potential confounder for self-management behavior. Third, the cross-sectional design of the study limits causal inferences. Therefore, future research should include prospective cohort and intervention studies to more thoroughly explore these dynamics. Longitudinal studies are particularly vital for providing definitive evidence on the causal effects of enhanced patient-centered care on long-term diabetes management. These studies will not only validate our current findings but also help refine intervention strategies to maximize their effectiveness in improving diabetes outcomes.

## Conclusions

The current study supports the significant association between perceived care quality and improved glycemic control in T2D, with self-care behaviors playing a central mediating role. The study delivers strategic insights that have the potential to transform T2D management by emphasizing patient-centered care and comprehensive self-care protocols, thus optimizing glycemic outcomes under China’s NEPHSP. These findings highlight the critical need for targeted patient education programs that enhance knowledge and engagement, problem-solving training to assist patients in managing daily challenges effectively, and robust follow-up and care coordination to ensure consistent care. Implementing these initiatives is crucial for amplifying the therapeutic effects of self-care behaviors and ultimately improving health outcomes for T2D patients.

### Electronic supplementary material

Below is the link to the electronic supplementary material.

Supplementary Material 1

## Data Availability

The datasets generated and/or analysed during the current study are available from the corresponding author on reasonable request.

## Abbreviations

NEPHSP: National Essential Public Health Services Program
PACIC: Patient Assessment of Chronic Illness Care
IQR: Interquartile range
OR: Odds Ratio
CI: Confidence interval
TE: Total effect
NDE: Natural direct effect
NIE: Natural indirect effect
PM: Percentage mediated

## References

[1] NCD Risk Factor Collaboration. Worldwide trends in diabetes since 1980: a pooled analysis of 751 population-based studies with 4.4 million participants. Lancet. 2016;387(10027):1513–30. 27061677 10.1016/S0140-6736(16)00618-8 PMC5081106

[2] Wang L, Peng W, Zhao Z, Zhang M, Shi Z, Song Z, et al. Prevalence and treatment of diabetes in China, 2013–2018. JAMA. 2021;326(24):2498–506. 34962526 10.1001/jama.2021.22208 PMC8715349

[3] Li Y, Teng D, Shi X, Qin G, Qin Y, Quan H, et al. Prevalence of diabetes recorded in mainland China using 2018 diagnostic criteria from the American Diabetes Association: national cross sectional study. BMJ. 2020;369:m997. 32345662 10.1136/bmj.m997 PMC7186854

[4] Xiong S, Jiang W, Meng R, Hu C, Liao H, Wang Y, et al. Factors associated with the uptake of national essential public health service package for hypertension and type-2 diabetes management in China’s primary health care system: a mixed-methods study. Lancet Reg Health West Pac. 2023;31:100664. 36879777 10.1016/j.lanwpc.2022.100664 PMC9985050

[5] Xiong S, Cai C, Jiang W, Ye P, Ma Y, Liu H, et al. Primary health care system responses to non-communicable disease prevention and control: a scoping review of national policies in Mainland China since the 2009 health reform. Lancet Reg Health West Pac. 2023;31:100390. 36879784 10.1016/j.lanwpc.2022.100390 PMC9985060

[6] You L, Zhao J, Chen X, Yang L, Liu M, Pan Y, et al. National Essential Public Health Services Programs over the past Decade Research Report two: progress and achievements of the lmplementation of National Essential Public Health Services Programs over the past Decade. Chin Gen Prac. 2022;25(26):3209–20. (Chinese).

[7] Li X, Lu J, Hu S, Cheng KK, De Maeseneer J, Meng Q, et al. The primary health-care system in China. Lancet. 2017;390(10112):2584–94. 29231837 10.1016/S0140-6736(17)33109-4

[8] Glasgow RE, Wagner EH, Schaefer J, Mahoney LD, Reid RJ, Greene SM. Development and validation of the Patient Assessment of Chronic Illness Care (PACIC). Med Care. 2005;43(5):436–44. 15838407 10.1097/01.mlr.0000160375.47920.8c

[9] Schmittdiel J, Mosen DM, Glasgow RE, Hibbard J, Remmers C, Bellows J. Patient Assessment of Chronic Illness Care (PACIC) and improved patient-centered outcomes for chronic conditions. J Gen Intern Med. 2008;23(1):77–80. 18030539 10.1007/s11606-007-0452-5 PMC2173922

[10] Fan J, McCoy RG, Ziegenfuss JY, Smith SA, Borah BJ, Deming JR, et al. Evaluating the structure of the Patient Assessment of Chronic Illness Care (PACIC) survey from the patient’s perspective. Ann Behav Med. 2015;49(1):104–11. 25236671 10.1007/s12160-014-9638-3

[11] Glasgow RE, Whitesides H, Nelson CC, King DK. Use of the Patient Assessment of Chronic Illness Care (PACIC) with diabetic patients: relationship to patient characteristics, receipt of care, and self-management. Diabetes Care. 2005;28(11):2655–61. 16249535 10.2337/diacare.28.11.2655

[12] Gugiu C, Coryn CL, Applegate B. Structure and measurement properties of the Patient Assessment of Chronic Illness Care instrument. J Eval Clin Pract. 2010;16(3):509–16. 20210824 10.1111/j.1365-2753.2009.01151.x

[13] Liu LJ, Li Y, Sha K, Wang Y, He X. Patient assessment of chronic illness care, glycemic control and the utilization of community health care among the patients with type 2 diabetes in Shanghai, China. PLoS ONE. 2013;8(9):e73010. 24039847 10.1371/journal.pone.0073010 PMC3769367

[14] Aung E, Donald M, Williams GM, Coll JR, Doi SA. Joint influence of patient-assessed chronic illness care and patient activation on glycaemic control in type 2 diabetes. Int J Qual Health Care. 2015;27(2):117–24. 25663097 10.1093/intqhc/mzv001

[15] Chinese Diabetes Society, National Office for Primary Diabetes Care. National guidelines for the prevention and control of diabetes in primary care (2022). Chin J Intern Med. 2022;61(3):249–62. (Chinese). 10.3760/cma.j.cn112138-20220120-000063 35263966

[16] American Diabetes Association. 5. Lifestyle Management: Standards of Medical Care in Diabetes-2019. Diabetes Care. 2019;42(Suppl 1):S46-s60. 10.2337/dc19-S005 30559231

[17] Bukhsh A, Khan TM, Nawaz MS, Ahmed HS, Chan KG, Lee LH, et al. Association of diabetes-related self-care activities with glycemic control of patients with type 2 diabetes in Pakistan. Patient Prefer Adherence. 2018;12:2377–85. 30519003 10.2147/PPA.S177314 PMC6235006

[18] Che M, Zhou Q, Lin W, Yang Y, Sun M, Liu X, et al. Healthy lifestyle score and Glycemic Control in type 2 diabetes Mellitus patients: a city-wide survey in China. Healthc (Basel). 2023;11(14):2037. 10.3390/healthcare11142037 PMC10379053 37510476

[19] van Houtum L, Heijmans M, Rijken M, Groenewegen P. Perceived quality of chronic illness care is associated with self-management: results of a nationwide study in the Netherlands. Health Policy. 2016;120(4):431–9. 27017047 10.1016/j.healthpol.2015.11.006

[20] Miao DD, Pan EC, Zhang Q, Sun ZM, Qin Y, Wu M. Development and validation of a Model for Predicting Diabetic Nephropathy in Chinese people. Biomed Environ Sci. 2017;30(2):106–12. 28292348 10.3967/bes2017.014

[21] Faul F, Erdfelder E, Buchner A, Lang AG. Statistical power analyses using G*Power 3.1: tests for correlation and regression analyses. Behav Res Methods. 2009;41(4):1149–60. 19897823 10.3758/BRM.41.4.1149

[22] Weykamp C. HbA1c: a review of analytical and clinical aspects. Ann Lab Med. 2013;33(6):393–400. 24205486 10.3343/alm.2013.33.6.393 PMC3819436

[23] Pang J, Zhang L, Li X, Sun F, Qiu J, Zhao Y, et al. Identification of factors associated with fear of hypoglycemia using the capability, opportunity, motivation and behavior model in people with type 2 diabetes mellitus: a cross-sectional study. Acta Diabetol. 2023;60(10):1405–15. 37380727 10.1007/s00592-023-02132-w

[24] Sun Z, Hu Y, Yu C. Low-risk lifestyle and health factors and risk of mortality and vascular complications in Chinese patients with diabetes. J Clin Endocrinol Metab. 2022;107(9):e3919–28. 35460564 10.1210/clinem/dgac264 PMC9387694

[25] Hu B, Lin LF, Zhuang MQ, Yuan ZY, Li SY, Yang YJ, et al. Reliability and relative validity of three physical activity questionnaires in Taizhou population of China: the Taizhou Longitudinal Study. Public Health. 2015;129(9):1211–7. 25957853 10.1016/j.puhe.2015.03.024

[26] Liu G, Li Y, Pan A, Hu Y, Chen S, Qian F, et al. Adherence to a healthy Lifestyle in Association with Microvascular complications among adults with type 2 diabetes. JAMA Netw Open. 2023;6(1):e2252239. 36701156 10.1001/jamanetworkopen.2022.52239 PMC9880795

[27] Chinese Preventive Medicine Association. Chinese guideline on healthy lifestyle to prevent cardiometabolic diseases. Chin J Prev Med. 2020;54(3):256–77. 10.3760/cma.j.issn.0253-9624.2020.03.006 32100977

[28] Zhang Y, Pan XF, Chen J, Xia L, Cao A, Zhang Y, et al. Combined lifestyle factors and risk of incident type 2 diabetes and prognosis among individuals with type 2 diabetes: a systematic review and meta-analysis of prospective cohort studies. Diabetologia. 2020;63(1):21–33. 31482198 10.1007/s00125-019-04985-9

[29] Zhang YB, Pan XF, Chen J, Cao A, Xia L, Zhang Y, et al. Combined lifestyle factors, all-cause mortality and cardiovascular disease: a systematic review and meta-analysis of prospective cohort studies. J Epidemiol Community Health. 2021;75(1):92–9. 32892156 10.1136/jech-2020-214050

[30] Limpens MAM, Asllanaj E, Dommershuijsen LJ, Boersma E, Ikram MA, Kavousi M et al. Healthy lifestyle in older adults and life expectancy with and without heart failure. 2022; 37: 37(2):205–14. 10.1007/s10654-022-00841-0 PMC8960597 35083603

[31] Pearl J. Interpretation and identification of causal mediation. Psychol Methods. 2014;19(4):459–81. 24885338 10.1037/a0036434

[32] Yung Y-F, Lamm M, Zhang W. Causal mediation analysis with the CAUSALMED procedure. Paper presented at: Proceedings of the SAS Global Forum 2018 Conference2018.

[33] MacKinnon DP, Fairchild AJ, Fritz MS. Mediation analysis. Annu Rev Psychol. 2007;58:593–614. 16968208 10.1146/annurev.psych.58.110405.085542 PMC2819368

[34] Heisler M, Smith DM, Hayward RA, Krein SL, Kerr EA. How well do patients’ assessments of their diabetes self-management correlate with actual glycemic control and receipt of recommended diabetes services? Diabetes Care. 2003;26(3):738–43. 12610031 10.2337/diacare.26.3.738

[35] Heisler M, Cole I, Weir D, Kerr EA, Hayward RA. Does physician communication influence older patients’ diabetes self-management and glycemic control? Results from the Health and Retirement Study (HRS). J Gerontol Biol Sci Med Sci. 2007;62(12):1435–42. 10.1093/gerona/62.12.1435 18166697

[36] Martorina W, Tavares A. Real-World Data in support of short sleep duration with poor Glycemic Control, in people with type 2 diabetes Mellitus. J Diabetes Res. 2019;2019:6297162. 31249843 10.1155/2019/6297162 PMC6556303

[37] Houle J, Beaulieu MD, Chiasson JL, Lespérance F, Côté J, Strychar I, et al. Glycaemic control and self-management behaviours in type 2 diabetes: results from a 1-year longitudinal cohort study. Diabet Med. 2015;32(9):1247–54. 25581545 10.1111/dme.12686

[38] Afroz A, Ali L, Karim MN, Alramadan MJ, Alam K, Magliano DJ, et al. Glycaemic Control for People with type 2 diabetes Mellitus in Bangladesh - An urgent need for optimization of management plan. Sci Rep. 2019;9(1):10248. 31308457 10.1038/s41598-019-46766-9 PMC6629620

[39] Ji M, Ren D, Dunbar-Jacob J, Gary-Webb TL, Erlen JA. Self-management behaviors, Glycemic Control, and metabolic syndrome in type 2 diabetes. Nurs Res. 2020;69(2):E9–17. 32108739 10.1097/NNR.0000000000000401

[40] Scarton L, Nelson T. Association of Medication Adherence with HbA1c Control among American Indian adults with type 2 Diabetes Using Tribal Health Services. Diabetes Care. 2023;46(6):1245–51. 37068266 10.2337/dc22-1885 PMC10234744

[41] Wang X, Strizich G, Hua S, Sotres-Alvarez D, Buelna C, Gallo LC, et al. Objectively measured sedentary Time and Cardiovascular risk factor control in US Hispanics/Latinos with Diabetes Mellitus: results from the Hispanic Community Health Study/Study of latinos (HCHS/SOL). J Am Heart Assoc. 2017;6(6):e004324. 28546455 10.1161/JAHA.116.004324 PMC5669141

[42] Behrens G, Fischer B, Kohler S, Park Y, Hollenbeck AR, Leitzmann MF. Healthy lifestyle behaviors and decreased risk of mortality in a large prospective study of U.S. women and men. Eur J Epidemiol. 2013;28(5):361–72. 23532745 10.1007/s10654-013-9796-9

[43] Al Mahrouqi AS, Mallinson RK, Oh KM, Weinstein AA. Patient-centred care, diabetes self-management and glycaemic control among Omani patients with type-2 diabetes. Int J Nurs Pract. 2023;29(1):e13103. 36045614 10.1111/ijn.13103

[44] Toobert DJ, Hampson SE, Glasgow RE. The summary of diabetes self-care activities measure: results from 7 studies and a revised scale. Diabetes Care. 2000;23(7):943–50. 10895844 10.2337/diacare.23.7.943

