# Supplementary material for: Associations between perceived care quality, self-care behaviors, and glycemic control in Chinese adults with type 2 diabetes under the national essential public health services program
Source: BMC Public Health. 2024 Jul 23;24:1964. doi: 10.1186/s12889-024-19538-y (PMC11265356; doi:10.1186/s12889-024-19538-y)
Supplement: Supplementary file 1 — Supplementary Material 1 [file 12889_2024_19538_MOESM1_ESM.docx]

**Associations Between Perceived Care Quality, Self-Care Behaviors, and Glycemic Control in Chinese Adults with** **Type 2 Diabetes under the National Essential Public Health Services Program**

**Supplementary Tables：**

**Supplementary Table S1. A summary of questionnair components: Comprehensive Research on the Prevention and Control of Diabetes (CRPCD) project (the part involved in this study)**

| **Individual questionnaire** | **Description** |
| --- | --- |
| Sociodemographic and clinical characteristics | 1.Gender: ① Male ②Female |
|  | 2.Date of birth: Year [ ] Month [ ] Day[ ] |
|  | 3.Education Level: ①Primary school or lower ②Junior school ③ Senior school or higher. |
|  | 4.Marital status: ①Married ②Single ③Separated ④Divorced ⑤ Widowed |
|  | 5.Occupational status: ①Employed ②Unemployed ③Retired. |
|  | 6.Annual household income (*thousand Yuan*): ① Less than 10 ② 10 to 100 ③ More than 100 |
|  | 7.During the past 12 months, did you take any glucose-lowering medications?  ①Yes ②No If No, go to 9 |
|  | 8. During the past 12 months, what types of glucose-lowering medications did you usually take?  ①Oral antihyperglycemic agents ②Insulin ③Both |
|  | 9.Has a doctor EVER told you that you had hypertension?  ①Yes ②No |
|  | 10.Has a doctor EVER told you that you had dyslipidemia?  ①Yes ②No |
|  | 1. 11.Has a doctor EVER told you that you have any of the following diseases? (You may select multiple options) 2. ①Nephropathy ②Retinopathy ③Neuropathy ④Coronary artery disease ⑤Stroke ⑥Peripheral arterial disease |
| Self-care behaviors | 12.In your life time, have you smoked a total of at least 100 cigarettes or equivalent? ①Yes ②No If No, go to 14 |
|  | 13.Have you smoked any tobacco today? ①Yes ②No |
|  | 14.During the past 12 months, how often did you drink any alcohol?  ①Never ②Only occasionally ③Only at certain seasons ④Every month but less than weekly ⑤Usually at least once a week If Never, go to 16 |
|  | 15.Have you drunk any alcohol today? ①Yes ②No |
|  | 16.During the past 12 months, about how often did you consume fruits?  ①Daily ②4-6 days per week ③1-3 days per week ④Monthly ⑤Never or rarely |
|  | 17.During the past 12 months, about how often did you consume vegetables?  ①Daily ②4-6 days per week ③1-3 days per week ④Monthly ⑤Never or rarely |
|  | 18.Do you do any vigorous-intensity sports, fitness or recreational (*leisure*) activities that cause large increases in breathing or heart rate like [*running or football,]* for at least 10 minutes continuously?   1. ①Yes ②No If No, go to 21 |
|  | 19.In a typical week, on how many days do you do moderate-intensity sports, fitness or recreational (*leisure*) activities?  Number of days [ ] |
|  | 20.How much time do you spend doing vigorous-intensity sports, fitness or recreational activities on a typical day?  Hours [ ] minutes [ ] |
|  | 21.Do you do any moderate-intensity sports, fitness or recreational *(leisure*) activities that causes a small increase in breathing or heart rate such as brisk walking*,*(*cycling, swimming, volleyball*)for at least 10 minutes continuously?  ①Yes ②No If No, go to 24 |
|  | 22.In a typical week, on how many days do you do moderate-intensity sports, fitness or recreational (*leisure*) activities?  Number of days [ ] |
|  | 23.How much time do you spend doing moderate-intensity sports, fitness or recreational (*leisure*) activities on a typical day?  Hours [ ] minutes [ ] |
|  | 24.How much time do you usually spend sitting or reclining on a typical day?  Hours [ ] minutes [ ] |
|  | 25.How much time do you typically sleep per day?  Hours [ ] minutes [ ] |
|  | 26.During the past 12 months, how often did you monitor your blood glucose?  ①Almost everyday ②At least once a week ③A few times a month ④A few times a year ⑤Less than once a year ⑥Never |
|  | 27.Do you ever forget to take your glucose-lowering medications?  ①Always ②Sometimes ③Never |
|  | 28.Have you been persuaded by non-medical personnel to alter your glucose-lowering regimen?  ①Always ②Sometimes ③Never |
|  | 27.Do you ever skip your glucose-lowering medications when you feel well?  ①Always ②Sometimes ③Never |
|  | 29.Have you adjusted your glucose-lowering medication in dosage or type due to illness without consulting a doctor?  ①Always ②Sometimes ③Never |

**Supplementary Table S2. Association of** **the overall PACIC score with individual self-care behaviors by using** **logistic regression models****(n=1,577)**

| **Self-care behaviors** | **Model 1** | |  | **Model 2** | |  | **Model 3** | |
| --- | --- | --- | --- | --- | --- | --- | --- | --- |
|  | OR (95% CI) | *P* value |  | OR (95% CI) | *P* value |  | OR (95% CI) | *P* value |
| No current smoking | 1.095(0.981,1.222) | 0.106 |  | 1.096(0.979,1.226) | 0.112 |  | 1.092(0.975,1.223) | 0.129 |
| No alcohol consumption | 1.117(0.993,1.257) | 0.064 |  | 1.124(0.986,1.283) | 0.081 |  | 1.111(0.972,1.269) | 0.122 |
| Healthy diet | 1.039(0.923,1.170) | 0.524 |  | 1.046(0.927,1.181) | 0.465 |  | 1.041(0.921,1.176) | 0.518 |
| Regular physical activity | 0.972(0.803,1.176) | 0.768 |  | 0.977(0.805,1.186) | 0.812 |  | 0.978(0.804,1.189) | 0.823 |
| Less sedentary behavior | 1.081(0.982,1.190) | 0.111 |  | 1.086(0.985,1.199) | 0.098 |  | 1.098(0.994,1.213) | 0.065 |
| Adequate sleep duration | 1.047(0.958,1.144) | 0.313 |  | 1.054(0.963,1.153) | 0.257 |  | 1.053(0.961,1.153) | 0.267 |
| Regular self-monitoring of blood glucose | 1.181(1.053,1.325) | 0.005 |  | 1.151(1.021,1.298) | 0.021 |  | 1.149(1.018,1.297) | 0.025 |
| Adequate medication adherence | 1.395(1.275,1.527) | <0.001 |  | 1.422(1.297,1.559) | <0.001 |  | 1.415(1.290,1.553) | <0.001 |
| Regular health check-ups | 1.750(1.591,1.926) | <0.001 |  | 1.756(1.594,1.936) | <0.001 |  | 1.775(1.609,1.958) | <0.001 |

Model 1: unadjusted.

Model 2: adjusted for age (continuous), gender, marital status, educational attainment, occupational status, annual household income, and glucose-lowering medication types.

Model 3: further adjusted for histories of hypertension, dyslipidemia, and the number of micro- and macrovascular complications.

*PACIC* Patient Assessment of Chronic Illness Care, *OR* odds ratio, *CI* confidence interval.

**Supplementary Table S3. Sensitivity analyses of mediation analyses for the association of** **the overall PACIC score and its subscales with HbA1c through the self-care behavior score**

| **PACIC** | **TE, β (95% CI)** | **NDE, β (95% CI)** | **NIE, β (95% CI)** | **PM, % (95% CI)** |
| --- | --- | --- | --- | --- |
| **Excluding participants who ceased smoking or drinking due to familial persuasion or financial constraints(n=1,517) ^a^** | | | | |
| Patient activation | -0.100(-0.172,-0.028)** | -0.057(-0.130,0.016) | -0.043(-0.061,-0.024)** | 42.76(8.07,77.45)* |
| Delivery system/practice design | -0.059(-0.150,0.031) | -0.013(-0.104,0.078) | -0.046(-0.066,-0.025)** | 77.29(-42.53,197.12) |
| Goal setting/tailoring | -0.071(-0.148,0.006) | -0.023(-0.100,0.055) | -0.048(-0.069,-0.028)** | 68.08(-8.76,144.93) |
| Problem-solving/contextual | -0.085(-0.160,-0.011)* | -0.039(-0.114,0.037) | -0.047(-0.066,-0.027)** | 54.71(3.01,106.42)* |
| Follow-up/coordination | -0.126(-0.202,-0.050)** | -0.080(-0.157,-0.003) | -0.046(-0.066,-0.026)** | 36.57(10.27,62.87)** |
| Overall PACIC score | -0.110(-0.194,-0.026)* | -0.055(-0.140,0.031) | -0.055(-0.078,-0.032)** | 49.95(7.52,92.38)* |
| **Excluding participants with micro- and macrovascular complications****(n=1,270) ^b^** | | | | |
| Patient activation | -0.098(-0.177,-0.018)* | -0.051(-0.131,0.029) | -0.047(-0.067,-0.026)** | 47.68(5.21,90.14)* |
| Delivery system/practice design | -0.063(-0.160,0.035) | -0.016(-0.114,0.081) | -0.047(-0.069,-0.024)** | 74.30(-42.80,191.39) |
| Goal setting/tailoring | -0.087(-0.171,-0.002)* | -0.034(-0.119,0.052) | -0.053(-0.076,-0.030)** | 61.06(-1.81,123.93) |
| Problem-solving/contextual | -0.101(-0.183,-0.019)* | -0.052(-0.135,0.031) | -0.049(-0.070,-0.027)** | 48.36(5.16,91.56)* |
| Follow-up/coordination | -0.136(-0.220,-0.052)** | -0.088(-0.173,-0.003) | -0.048(-0.070,-0.026)** | 35.30(9.31,61.30)** |
| Overall PACIC score | -0.120(-0.212,-0.028)* | -0.062(-0.156,0.031) | -0.058(-0.083,-0.032)** | 48.03(7.17,88.88)* |

^a^ Adjusted for age (continuous), gender, marital status, educational attainment, occupational status, annual household income, glucose-lowering medication types, history of hypertension, history of dyslipidemia, and the number of micro- and macrovascular complications.

^b^ Adjusted for age (continuous), gender, marital status, educational attainment, occupational status, annual household income, glucose-lowering medication types, and histories of hypertension and dyslipidemia.

*PACIC* Patient Assessment of Chronic Illness Care, *TE* total effect, *NDE* natural direct effect, *NIE* natural indirect effect, *PM* percentage mediated, *CI* confidence interval

* *P* <0.05; ** *P* <0.01
